# The Decline in the Tuberculosis Death-Rate

**Published:** 1914-01-24

**Authors:** 


### The Hospital

**The Workers' Newspaper of Administrative Medicine and Institutional Life, Administration, National Insurance and Health.**

No. 1439, Vol. LV. SATURDAY, JANUARY 24, 1914. PRICE ONE PENNY

### THE DECLINE IN THE TUBERCULOSIS DEATH-RATE.

While very, very few deny that tuberculosis has declined in the last thirty years, certainly many, when making the admission, will add also qualifications. They will say that in all probability the disease had been lessening for a period much longer. They may deprecate any assumption of credit for this phenomenon by sanitarians, and may point to Aberdeen, which city, until lately without concerted anti-tuberculosis measures, can show a fall in the consumption death-rate more marked than that in Edinburgh, which is, of course, the headquarters of the now familiar and fashionable "dispensary" movement. It is admitted, too, that the few years following, as compared with the few years preceding, Koch's identification of the causal micro-organism of tuberculosis, did not show any resulting benefit to mankind in the diminution of the scourge. But explain the thing how one will, there is no more doubt that considerably less consumption exists than used to be, than that there is vastly less smallpox under George V. than under George IV. And so—let us commend this point to certain obscurantists—here is something proved to general satisfaction by statistics alone.

But, although the broad fact itself emerges, many of its constituent particulars remain highly uncertain, inscrutable, mysterious, and inter-contradictory; and it is reassuring to find a full appreciation of this in any inquiry directed towards the elucidation of these same particulars. A paper* by Dr. Hoffman, which shows a standard of accomplishment higher than that of most American medical articles, fitly opens with a confession of the difficulties involved in any detailed examination of the tuberculosis death-rate, more particularly in comparing the tuberculosis death-rate of one country with that of another. He defines, a little ambitiously, his object as being—"to set forth, with the required brevity, the results of an elaborate inquiry into the entire subject of tuberculosis statistics, and without any reference to any one of the various medical controversies, with regard to which the statistical expert has no concern." Let us emulate the author, and similarly "set forth with the required brevity" his main results. To begin with, he does not find that paradoxical excess in tuberculosis of rural over urban areas, which has been stated in one or two smaller examinations in this country. In America, for the decade ending 1909, the cities of the registration States showed a consumption death-rate of 177.4, while the figure for rural areas was 124.1. This, too, in spite of the fact that "there is a tendency for tuberculosis sanatoria to be established in country districts, and many rural sections show an excessive death-rate from tuberculosis entirely on that account." Next, although the difference in the tuberculosis death-rates of Great Britain and of America is only slight (and, therefore, considering the above-mentioned fallacies, probably negligible), yet four times as many Englishmen as Americans die from bronchitis. This latter phenomenon the author attributes to that general scapegoat, the British climate; but, as he also says, it merits collective investigation.

But the most taking part of Dr. Hoffman's paper comes at the end. He gives figures showing that in 1912 the tuberculosis death-rate was smaller than at any previous time of which proper demographical record exists. But, unlike some others, he is not moved thereby to any rash prophecy, which really seems an indulgence especially dangerous in any department of phthisiology. There have been in these last few years many pronouncements by bacteriologists, who apparently look upon tuberculosis as, as much an infectious disease as plague or scarlatina, and by sanitarians and publicists inspired by them, promising freely the abolition of tuberculosis in a few decades at most. Dr. Hoffman is not one of these rash people. He points out that, although a certain diminution in mortality may readily happen and be readily furthered, as the result of certain measures, yet commonly an obstinate residuum of cases persists; to lessen that residuum demands huge efforts, just as to obtain an extra knot of speed in a new Atlantic liner. We would express entire agreement. So long, too, as a Northern climate makes people prefer for half the year rooms warmed and curtained to rooms freely pervaded by chilly

* *The Decline in the Tuberculosis Death-Rate*, 1871-1912. By F. L. Hoffman, LL.D. Newark, N.J.

external air, so long as weakly subjects neglect the laws of health, and so long as mental patients continue to show their marked proneness to tubercle, so long shall we meet with that disease. The corollary may even be drawn that a certain amount of it—of the human type of infection, that is, not the bovine, which cripples children—is, to a civilised race dwelling in the temperate zone, somewhat of an eugenic agency. This, however, is going beyond Dr. Hoffman's limits. We can recommend this pamphlet as well worth its half-inch of space on any tuberculosis worker's bookshelves.

